# Hepatitis B in Africa Collaborative Network: cohort profile and analysis of baseline data

**DOI:** 10.1017/S095026882300050X

**Published:** 2023-04-03

**Authors:** Nicholas Riches, Michael Vinikoor, Alice Guingane, Asgeir Johannessen, Maud Lemoine, Philippa Matthews, Edith Okeke, Yusuke Shimakawa, Roger Sombie, Alexander Stockdale, Gilles Wandeler, Monique Andersson, Pantong Davwar, Hailemichael Desalegn, Mary Duguru, Fatou Fall, Tongai Maponga, David Nyam Paul, Moussa Seydi, Edford Sinkala, Jantjie Taljaard, Mark Sonderup, C. Wendy Spearman

**Affiliations:** 1 Department of Clinical Sciences and International Public Health, Liverpool School of Tropical Medicine, Liverpool, UK; 2 Department of Internal Medicine, University of Zambia, Lusaka, Zambia; 3 Hepato-Gastroenterology Department, Bogodogo University Hospital Center, Ouagadougou, Burkina Faso; 4 Institute of Clinical Medicine, University of Oslo, Oslo, Norway; 5 Department of Metabolism, Digestion and Reproduction, Imperial College London, London, UK; 6 Nuffield Department of Medicine, University of Oxford, Oxford, UK; 7 Faculty of Medical Sciences, University of Jos, Jos, Nigeria; 8 Epidemiology of Emerging Diseases Unit, Pastor Institute, Paris, France; 9 Département d’Hépato-gastroentérologie, Yalgado Ouédraogo University Hospital Center, Ouagadougou, Burkina Faso; 10 Department of Clinical Infection, Microbiology and Immunology, Institute of Infection, Veterinary and Ecological Sciences, University of Liverpool, Liverpool, UK; 11 Institute of Social and Preventive Medicine, University of Bern, Bern, Switzerland; 12 Division of Medical Virology, Stellenbosch University Faculty of Medicine and Health Sciences, Cape Town, South Africa; 13 Medical Department, St. Paul’s Hospital Millennium Medical College, Addis Ababa, Ethiopia; 14 Department of Hepatology and Gastroenterology, Dakar Main Hospital, Dakar, Senegal; 15 Division of Medical Virology, Faculty of Medicine and Health Sciences, Stellenbosch University, Cape Town, South Africa; 16 Department of Infectious and Tropical Diseases, Regional Center for Research and Training, Fann National University Hospital Center, Dakar, Senegal; 17 Division of Infectious Diseases, Department of Medicine, Tygerberg Hospital and Stellenbosch University, Cape Town, South Africa; 18 Division of Hepatology, Department of Medicine, Faculty of Health Sciences, University of Cape Town, Cape Town, South Africa

**Keywords:** Epidemiology, hepatitis B, Africa, liver cirrhosis, hepatocellular carcinoma, antiviral agents

## Abstract

Approximately 80 million people live with chronic hepatitis B virus (HBV) infection in the WHO Africa Region. The natural history of HBV infection in this population is poorly characterised, and may differ from patterns observed elsewhere due to differences in prevailing genotypes, environmental exposures, co-infections, and host genetics. Existing research is largely drawn from small, single-centre cohorts, with limited follow-up time. The Hepatitis B in Africa Collaborative Network (HEPSANET) was established in 2022 to harmonise the process of ongoing data collection, analysis, and dissemination from 13 collaborating HBV cohorts in eight African countries. Research priorities for the next 5 years were agreed upon through a modified Delphi survey prior to baseline data analysis being conducted. Baseline data on 4,173 participants with chronic HBV mono-infection were collected, of whom 38.3% were women and the median age was 34 years (interquartile range 28–42). In total, 81.3% of cases were identified through testing of asymptomatic individuals. HBeAg-positivity was seen in 9.6% of participants. Follow-up of HEPSANET participants will generate evidence to improve the diagnosis and management of HBV in this region.

## Introduction

Viral hepatitis is a growing cause of global morbidity and mortality, with most deaths attributed to chronic hepatitis B virus (HBV) infection [1]. HBV is an efficient human pathogen, with approximately 316 million people worldwide having chronic HBV infection, which can cause progressive liver disease and ultimately death due to complications of cirrhosis and/or hepatocellular carcinoma (HCC) [1, 2]. HBV-related disease caused 555,000 deaths in 2019, and HBV-related mortality is anticipated to continue to grow with the ageing of populations in high HBV prevalence settings, such as the Western Pacific and Africa [2].

As part of the global hepatitis elimination agenda, targets have been set for HBV, including reducing incidence by 90% and mortality by 65% by 2030 compared with the 2015 baseline [3]. HBV mortality will only be reduced by making progress in diagnosing, linking to care, and treating people living with chronic HBV infection with antiviral therapies. Underdiagnosis of HBV is a major challenge, with less than 10% of people living with chronic HBV infection worldwide being aware of their infection [3, 4]. Although current therapies lower the risk of liver-related mortality, they seldom achieve ‘functional cure’ (i.e. loss of hepatitis B surface antigen), and thus most patients require lifelong therapy. There are several promising new therapeutic agents in development to achieve HBV functional cure. These will target HBV functional cure through a possible combination of antivirals, vaccine therapy, and immunotherapy [5, 6]. The new therapeutics, if effective and affordably implemented, could accelerate HBV elimination [7].

To achieve the global elimination of HBV, it is critical that Africa be designated a priority region, similar to HIV. In 2019, the WHO Africa Region had 82.3 million people living with chronic HBV infection including an estimated 990,000 (95% confidence interval (CI) 660,000–1,600,000) new chronic infections, corresponding to two-thirds of all new infections globally [8]. However, data on HBV natural history, diagnostics, treatment effects, and prevention strategies in Africa are sorely lacking. Nearly all published studies on HBV from the region merely describe cross-sectional prevalence estimates. Longitudinal data are limited to a handful of small and/or single country studies. HBV-related HCC in Africa is only described among hospitalised patients [9], and progression and response to therapy are not well documented in the literature. Some have argued that HBV is indeed a neglected tropical disease in Africa to draw attention to these knowledge gaps [10].

The epidemiology and natural history of HBV infection in Africa differ from other regions, and both these differences and co-factors may impact diagnostics, treatment effects, and prevention measures. HBV genotypes that are circulating in Africa (A1, D, and E) are generally less well characterised clinically, especially genotype E [11], compared with those dominating in the Asia-Pacific region (B and C). There are also unique environmental factors that intersect with HBV, including traditional medicines, khat use, which is a recognised cause of cirrhosis in East Africa [12], home-made alcohol brews augmenting iron overload [13], and exposure to aflatoxins which accumulate in stored maize and other foodstuffs [14]. Africa also has a high burden of endemic co-infections that may alter HBV natural history including schistosomiasis and malaria. Anti-tuberculosis medications, used for latent and active tuberculosis, are associated with drug-induced liver injury. HIV is a major public health issue in Africa and may modulate HBV infection in important ways, accelerating the progression of liver disease, and increasing HBV viral load and risk of transmission [15]. Non-alcoholic fatty liver disease is an emerging liver disease co-factor [16, 17]. As has been seen in hepatitis C, there are also likely to be unique host genetic and immunologic factors in Africa that alter the immune response to HBV and propensity for HCC [18, 19].

To close knowledge gaps around HBV in Africa, we established the multi-country HBV research collaboration ‘Hepatitis B in Africa Collaborative Network’ (HEPSANET; https://www.hepsanet.org). The central mission of HEPSANET is to collect, combine, analyse, and disseminate data to inform regional HBV policy and clinical care. In this paper, we describe HEPSANET’s origin, procedures, scientific priorities, and baseline results.

## Materials and methods

### Origin of the collaboration

The neglect of HBV in Africa as a research and public health topic led to the creation of a small network of clinicians and researchers over the past decade. This network formed organically with facilitation from international liver conferences. As individual sites and research teams have implemented their projects in East, Southern, and West Africa, it became clear that multi-country and multi-regional approaches would be needed to address the limitations of single-site cohort studies. Therefore, following the 2020 Conference for Liver Diseases in Africa, a group of investigators decided to formally create a collaboration to share data, and HEPSANET was established. A systematic review was conducted to identify additional research groups, and, within weeks, many major groups working on HBV in Africa were invited and joined. During a series of conference calls, the general framework was discussed and agreed upon. A data-sharing agreement was created and signed by all parties.

### Cohort participation

At inception, included cohorts were those managed by many of the most active researchers and treatment centres working on HBV across Africa. Most cohorts are managed from urban and university hospitals, but some are population-based having a catchment area that includes rural populations. Most have local access to HBV DNA testing and elastography for non-invasive assessment of liver fibrosis and cirrhosis. Many also have access to abdominal ultrasound and CT scan for the diagnosis of HCC. Most cohorts participating in HEPSANET had already published their HBV data in peer-reviewed journals.

At each cohort, treatment of HBV is initiated according to national guidelines and/or international guidelines from the World Health Organization (WHO) (2015) [20], European Association for the Study of the Liver (EASL) (2017) [21], and American Association for the Study of the Liver (2016) [22]. For adults and adolescents, treatment consists of tenofovir disoproxil fumarate or tenofovir alafenamide and occasionally lamivudine. For those in countries with large HIV programmes, patients with HBV are prescribed coformulations of tenofovir with lamivudine or emtricitabine.

### Ethical considerations

Investigators participating had local country ethical approvals to collect and publish their data and to share de-identified data publicly. These approvals were shared with HEPSANET for inclusion whenever necessary during data dissemination (i.e. as part of journal publication). All data were stripped of any personal health identifiers (names, address, etc.) at the cohort level, and only de-identified data were shared.

### Consortium organisation and governance

HEPSANET has a rotating secretariat. The role of the secretariat is to organise meetings, provide a data report, track analyses and manuscripts in development and submission, maintain regulatory approvals, and coordinate other issues that arise. Meetings occur virtually on a bimonthly basis, with additional in-person meetings at major international liver and infectious diseases conferences. Documents and recordings from meetings are kept in a cloud-based server to permit sharing among investigators and allow for smooth transfer of secretariat responsibility. Within HEPSANET, working groups of 3–5 investigators were created to accelerate progress towards addressing the scientific agenda. This includes groups for data harmonisation and management, epidemiology and testing, liver endpoints including HCC, and prevention of mother-to-child transmission. Working groups brief the wider group at bimonthly meetings. Discussions are also underway to include civil society input, including the establishment of a steering group of people living with chronic HBV infection, which can help inform and shape research and dissemination strategies.

### Development of scientific agenda

To define HEPSANET’s scientific agenda, we used a three-round modified Delphi process to identify and rank research priorities [23, 24]. All stages were conducted online using a Google form. For the first round, investigators were each asked to ‘list up to 10 research priorities for HEPSANET for the next 5 years’. In the second round, investigators were asked to assign a priority score to each priority from 1 (low priority) to 5 (high priority). Priorities with the highest scores were selected, and we, then, undertook a final round to clarify consensus over the ranking.

### Inclusion and exclusion criteria

HEPSANET used very inclusive definitions for data to be shared by cohorts. The initial sample included HBsAg-positive people who were aged 13 years and older and had chronic HBV infection. Each analysis, then, implemented various exclusion criteria, such as prior antiviral therapy, the presence of HIV, hepatitis C, or hepatitis delta coinfection.

### Data collection, harmonisation, and quality assurance

A range of variables are collected by HEPSANET, including demographic factors, HBV testing and treatment history, and clinical parameters (see Supplementary Table 1). For longitudinal time points, HEPSANET requests a cohort outcome variable for each patient. Outcome options include: retained in cohort, transferred out (patient relocated to distant area or left cohort for another HBV clinic), withdrew (patient left the cohort), died, and lost to follow-up (LTFU). LTFU is defined in diverse ways across sites, but for HEPSANET, it is defined as >12 months late for a scheduled visit and prior to the data censoring date (i.e., the date when the data were uploaded). Each participating cohort independently collects its own data and is responsible for creating datasets that are de-identified (no names, contact information, etc.) and standardised using a standard operating procedure that carefully defines each variable. Then cohort-level datasets are uploaded to a cloud-based system, and a data manager appends the data. The first data upload occurred in 2021, and additional uploads are expected annually. Following each new data upload, a data manager generates a summary on data availability, including missingness, and cohorts are asked to clarify issues that are identified and, as necessary, re-submit corrected data.

### Statistical analysis of baseline characteristics of HEPSANET

To provide a description of HEPSANET data, we analysed the characteristics of participants at baseline, defined as the earliest available data for each participant. This analysis is restricted to participants aged 18 and over without comorbid HIV infection. Stratified by region, we displayed baseline demographics, HBV treatment history, and key clinical parameters. We described HBV testing patterns (i.e. how participants learned their status) and made comparisons by region and by sex. Among treatment-naïve participants with baseline ALT, HBV DNA, and hepatitis B e-antigen (HBeAg), we described the range of HBV phenotypes represented in HEPSANET, based on EASL categories [21]. ALT elevation was defined as ≥30 for men and ≥ 19 for women, as suggested in the WHO guidelines [20]. We classified participants who could not be categorised based on their baseline results as having an ‘indeterminate’ phenotype.

We then explored differences in HBV natural history by the HEPSANET region. The West African region included participants from Burkina Faso, Gambia, Nigeria, and Senegal. East African data came from Ethiopia, whereas Southern African data covered Malawi, South Africa, and Zambia. Patients diagnosed on clinical suspicion of HBV infection were excluded as we previously found that they are enriched for cirrhosis and elevated markers of HBV replication [25]. Participants not currently on anti-HBV treatment and who learnt of their HBV status through asymptomatic testing were assessed by comparing markers of HBV replication (HBV DNA and HBeAg) and of possible liver disease (ALT and median liver stiffness measurement (LSM); based on elastography). HBV DNA was dichotomised at 2,000 IU/ml, and ALT was dichotomised as normal or elevated. LSM was dichotomised at ≥9.5 kPa, a threshold reported for cirrhosis in West Africa [26]. Comparisons between regions were done using multivariable logistic regression with West Africa as the reference group and were adjusted for age, sex, and the type of asymptomatic testing. Asymptomatic testing types were categorised as either targeted testing (testing through blood transfusion services, antenatal clinics, occupational healthcare, or for work, marriage, or travel) or population-based screening.

## Results

### Scientific priorities

During the first round of the modified Delphi approach, 13 HBV expert respondents generated a list of 104 potential priorities in the area of HBV in the WHO African Region Africa. In the second round, 15 respondents assigned scores to each listed priority. From there, 11 priorities had the top 10 scores (two were tied for the 10th position). In a final round, 11 respondents gave an ordered rank of these priorities. The final list of HEPSANET’s 10 highest-ranked objectives is displayed in Table 1.

**Table 1.** HEPSANET scientific agenda: highest priority objectives

Rank	Research objective
1	Characterise the natural history of chronic HBV infection in longitudinal research (including virological, serological, and clinical outcomes)
2	Develop simplified HBV treatment eligibility criteria for Africa
3	Evaluate strategies to integrate HBV testing and treatment into existing health systems
4	Evaluate novel approaches to diagnose HCC
5	Evaluate scalable screening strategies for HCC in people with hepatitis B
6	Measure the cost and cost-effectiveness of different HBV testing strategies
7	Investigate the public health benefits of HBV therapy (i.e. treatment as prevention)
8	Involve African populations in research into novel HBV therapeutics and therapeutic strategies
9	Strengthen the evidence base for HBV birth dose vaccine
10	Evaluate novel point-of-care tests for HBV

Abbreviation: HBV, hepatitis B virus; HCC, hepatocellular carcinoma; HEPSANET, Hepatitis B in Africa Collaborative Network.

### Baseline demographics and clinical characteristics

**Figure 1.**
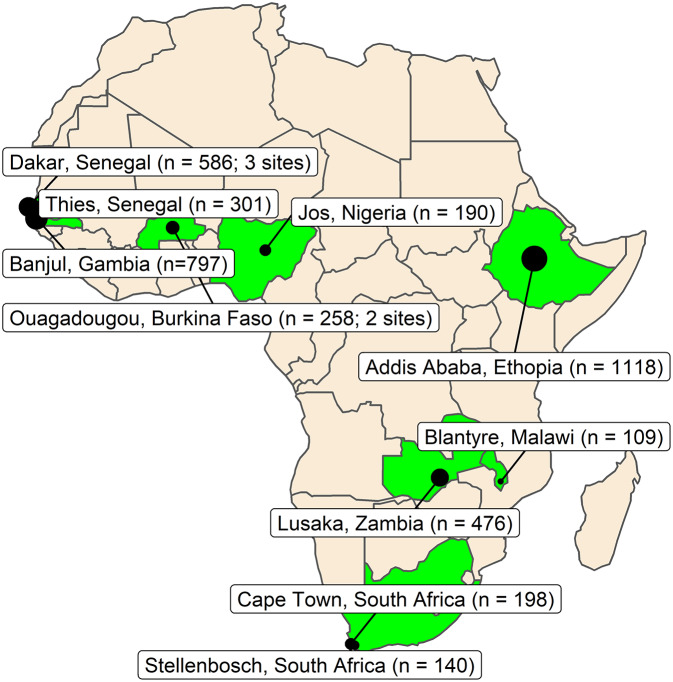
Map of included studies. *Note*: *n* is the number of hepatitis B participants included from each site at baseline. The area of the circles is proportionate to the cohort size

By 1 June 2022, 13 cohorts had formally entered HEPSANET and shared data, from across eight countries and three regions of Africa, creating a pooled dataset of 4,173 unique participants with chronic HBV infection (Fig. 1).

We described the characteristics of HEPSANET participants at baseline, stratified by region (Table 2). Excluding missing data, 38.8% of the sample were women, the median age was 34 years (interquartile range (IQR) 28–42), 31.3% were less than 30 years old, and 12.0% were over 50 years old. A total of 3,941 people had a documented treatment status, of whom 3,772 (95.7%) were not on treatment enrolment. The median body mass index (BMI) of participants was 22.4 (IQR 20.0–25.6), and 229 (6.7%) were obese (BMI ≥ 30 kg/m^2^). HBeAg-positivity was present in 338 participants (9.6%). The median HBV DNA level was 573 IU/ml (IQR 59–5,688), and the majority had HBV DNA <2,000 IU/ml (*n* = 2,231, 64.3% of those with valid measurements). Transient elastography results were available for 3,892 (93.3%). Among them, 556 (14.3%) had cirrhosis based on LSM ≥9.5 kPa. Unhealthy alcohol use was reported among 274 (9.0%) of the 3,031 assessed.

**Table 2.** Summary of baseline demographic and clinical data from HEPSANET cohorts

Characteristics *n* (%) or median (IQR)	Valid observations (*n*)	West Africa (*n* = 2,132)	East Africa (*n* = 1,118)	Southern Africa (*n* = 923)
*Demographics*				
Age (median)	4,173	34.0 (29.0, 42.0)	32.0 (26.0, 39.0)	35.0 (28.0, 43.0)
Age group	4,173			
18–29		593 (27.8%)	442 (39.5%)	271 (29.4%)
30–49		1,277 (59.9%)	582 (52.1%)	507 (54.9%)
50+		262 (12.3%)	94 (8.4%)	145 (15.7%)
Sex	4,172			
Female		800 (37.5%)	455 (40.7%)	366 (39.7%)
Male		1,332 (62.5%)	663 (59.3%)	556 (60.3%)
Reason for testing	3,740			
Focused asymptomatic testing		1,153 (60.9%)	750 (67.1%)	306 (41.9%)
Community-based screening		603 (31.9%)	0 (0.0%)	230 (31.5%)
Suspicion of liver disease		78 (4.1%)	368 (32.9%)	180 (24.7%)
Family member with liver disease		26 (1.4%)	0 (0.0%)	13 (1.8%)
Other		32 (1.7%)	0 (0.0%)	1 (0.1%)
Current HBV treatment	4,173			
Yes		12 (0.6%)	29 (2.6%)	128 (13.9%)
No		1,891 (88.7%)	1,089 (97.4%)	792 (85.8%)
Not documented		229 (10.7%)	0 (0.0%)	3 (0.3%)
*Diagnostic test results*				
TE score (median, kPa)	3,892	5.4 (4.5, 6.8)	5.8 (4.6, 7.7)	6.1 (4.8, 8.5)
TE score group	3,892			
<8 kPa		1,743 (83.8%)	857 (76.7%)	491 (70.9%)
8.5–9.5 kPa		128 (6.2%)	54 (4.8%)	63 (9.1%)
≥9.5 kPa		210 (10.1%)	207 (18.5%)	139 (20.1%)
ALT (IU/ml)	4,103	24.0 (18.0, 33.0)	25.0 (19.0, 36.0)	25.0 (17.0, 38.2)
AST (IU/ml)	4,058	28.0 (23.0, 35.0)	25.5 (20.0, 34.0)	29.0 (22.0, 43.5)
Platelet count	3,834	216.0 (171.0, 265.2)	277.0 (225.0, 329.0)	219.0 (170.8, 274.0)
HBeAg status	3,539			
Positive		94 (5.2%)	114 (11.3%)	130 (17.8%)
Negative		1,706 (94.8%)	895 (88.7%)	600 (82.2%)
HBV DNA level	3,469	231.0 (50.0, 2,518.0)	1,239.0 (245.0, 12,571.0)	743.0 (59.0, 12,991.1)
*Comorbid conditions*				
Hazardous alcohol use	3,031			
Yes		128 (11.2%)	40 (3.6%)	106 (13.7%)
No		1,010 (88.8%)	1,078 (96.4%)	669 (86.3%)
BMI (median)	3,443	22.3 (20.1, 25.5)	22.4 (19.6, 25.3)	22.5 (20.4, 26.1)
BMI group	3,443			
Underweight (<18)		119 (6.7%)	103 (9.9%)	44 (7.1%)
Normal weight (18–24)		1,152 (64.7%)	648 (62.4%)	388 (62.3%)
Overweight (25–29)		394 (22.1%)	230 (22.1%)	136 (21.8%)
Obese (≥30)		116 (6.5%)	58 (5.6%)	55 (8.8%)

Abbreviations: BMI, body mass index; HBeAg, hepatitis B e-antigen; HBV, hepatitis B virus; HEPSANET, Hepatitis B in Africa Collaborative Network; IQR, interquartile range; IU/ml, international units per millilitre; kPA, kilopascals; TE, transient elastography.

### Reasons for HBV testing

**Figure 2.**
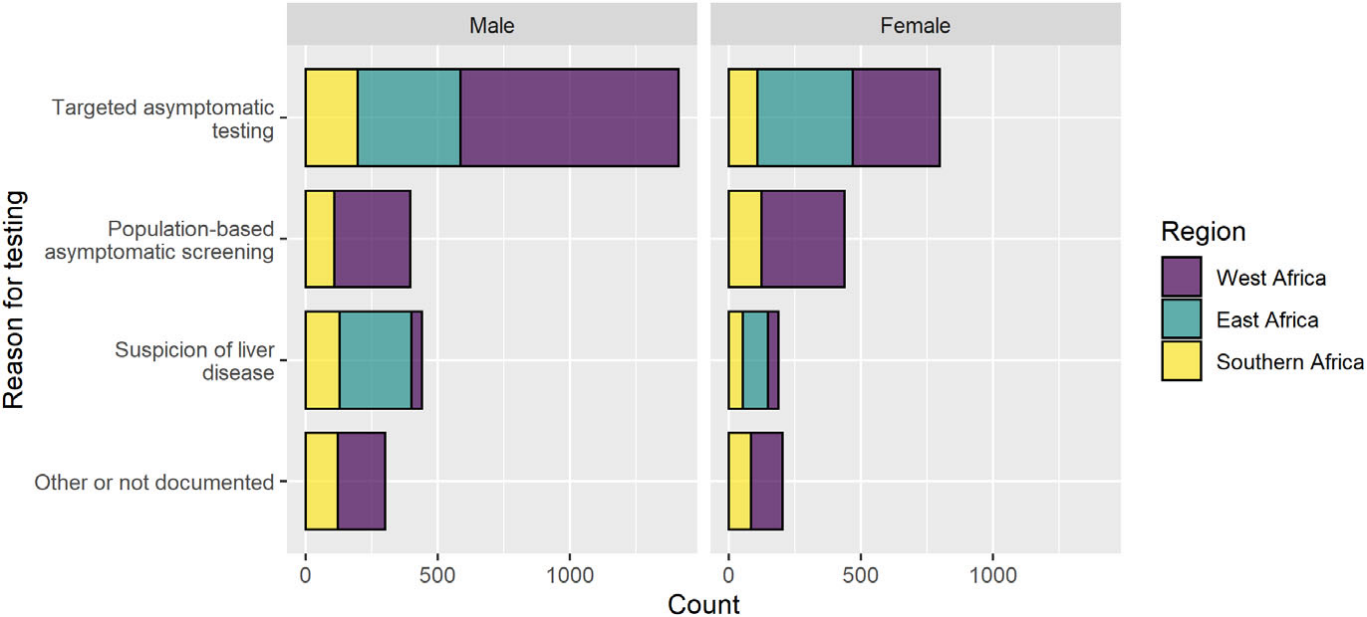
Reason for hepatitis B testing in HEPSANET cohorts, stratified by sex and region. Abbreviation: ANC, antenatal clinic

At baseline, most (78.2%) HEPSANET cohorts were managed through hospital-based clinics in urban settings. The majority (81.3%) of participants were asymptomatic and learned their HBV status through testing at a routine screening venue (Fig. 2). This included population-based screening programmes (22.3%) and targeted testing done at the blood bank, during antenatal care visits or a routine medical check-up for travel, marriage, or a job (59.1%). Diagnosis through a population-based screening programme was most common in West African cohorts. While most were asymptomatic when they learned their HBV status, a minority (16.7%) of participants in HEPSANET were diagnosed when they presented to a health facility with signs or symptoms of liver disease, such as jaundice or decompensated liver disease. Diagnosis on clinical suspicion was most common in East Africa (32.9%) and least common in cohorts from Southern (24.7%) and West (4.1%) Africa. Across HEPSANET, women were slightly more likely to learn their status via asymptomatic screening than men (85.0% vs. 79.0%; *P* < 0.001). A slightly larger proportion of men, compared with women, learned their HBV status when presenting with signs or symptoms (19.2% vs. 12.8%; *P* < 0.001).

### Hepatitis B phenotypes

**Figure 3.**
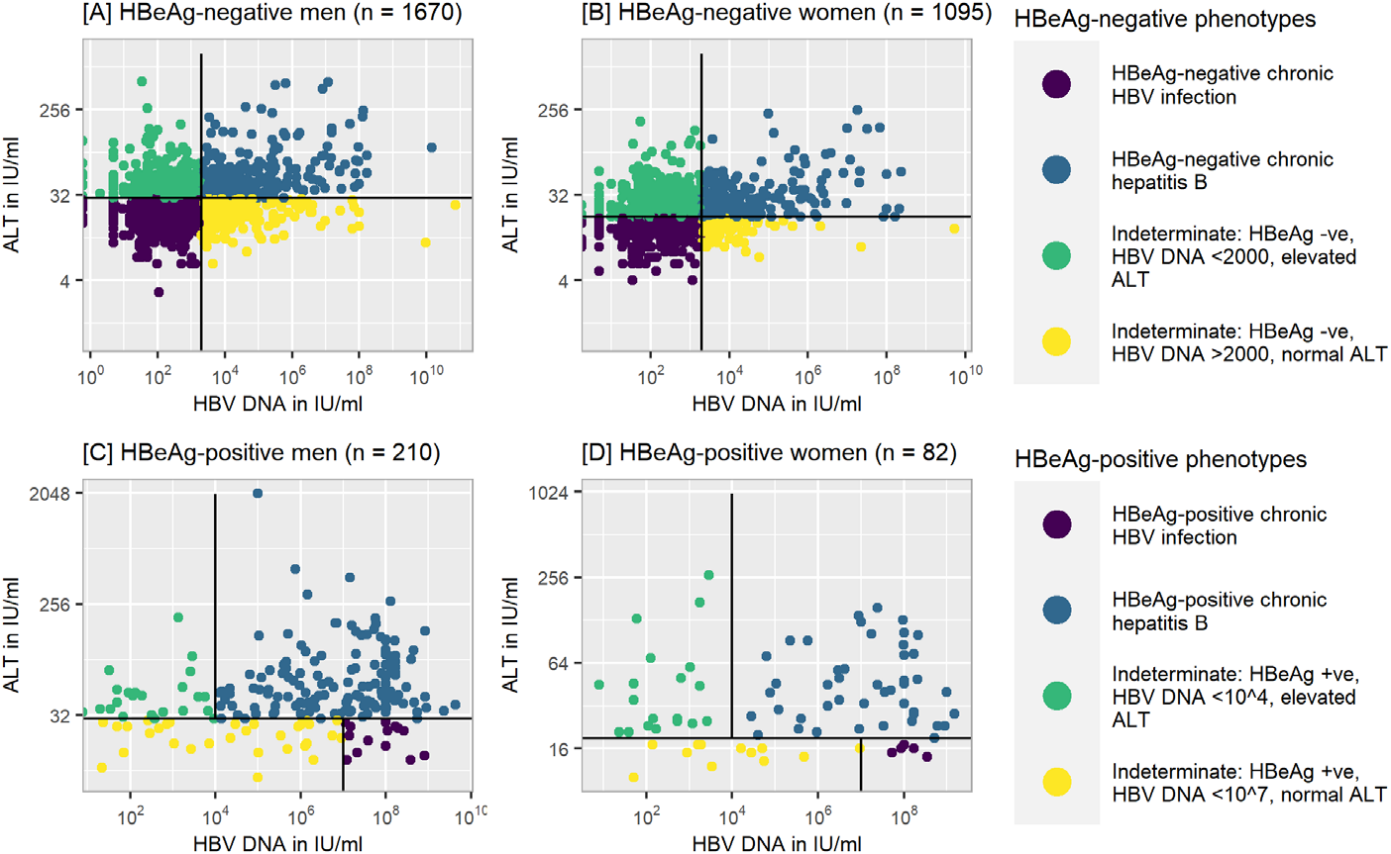
Hepatitis B phenotypes, stratified by HBeAg status and sex. *Note*: Analysis performed on a subset of participants (*n* = 3,057) with valid data on sex and laboratory data, and not currently on treatment

Among HEPSANET participants with chronic HBV infection, not currently on treatment, and not missing data for HBeAg, HBV DNA, and ALT, a range of HBV phenotypes was present (Fig. 3). HBeAg-positive chronic HBV infection (also referred to as ‘immune tolerant state’) was rare, seen in only 0.4% of women and 0.7% of men. Chronic hepatitis B (i.e. elevation of both HBV DNA and ALT) was present in 692 (22.6%) of participants. Most participants with chronic hepatitis B (72.8%) were HBeAg-negative. We classified 1,060 (34.7%) as having HBeAg-negative chronic HBV infection (commonly referred to as ‘immune control’). While most individuals could be categorised, 42.1% of participants had an indeterminate phenotype at baseline. The most common indeterminate phenotype, seen for 841 (27.5%), was HBeAg-negativity, low HBV DNA (<2,000 IU/ml), and elevated ALT. Less common, seen in 360 (11.8%), were participants who were HBeAg-negative, had HBV DNA >2,000 IU/ml, and a normal ALT.

### Regional differences in markers of HBV replication and liver disease

Among participants not currently taking HBV treatment and who were asymptomatic when learning their status, we explored differences by region in markers of HBV replication and liver disease (Table 3). The odds of having a positive HBeAg result (adjusted odds ratio (AOR) 2.81; 95% CI 1.91–4.12; *P* < 0.001) and having HBV DNA >2,000 IU/ml (AOR 1.98; 95% CI 1.57–2.49; *P* < 0.001) were increased in Southern Africa, considering West Africa as the reference group. The adjusted odds of cirrhosis (≥9.5 kPa) were lower in East (AOR 0.46; 95% CI 0.30–0.71; *P* < 0.001) compared with West Africa. We did not see any statistically significant differences in the odds of ALT elevation between regions.

**Table 3.** Adjusted odds ratios for age, sex, region, and testing type, predicting (i) HBeAg-positivity; (ii) elevated HBV DNA; (iii) elevated ALT; and (iv) elevated TE

Characteristics	(i) HBeAg +ve		(ii) Elevated HBV DNA^a^		(iii) Elevated ALT^b^		(iv) Cirrhosis by TE^c^	
	OR	*P*-value	OR	*P*-value	OR	*P*-value	OR	*P*-value
Age group								
18–29	—		—		—		—	
30–49	0.33 (0.23–0.47)	<0.001	0.65 (0.54–0.78)	<0.001	1.07 (0.90–1.27)	0.5	1.40 (0.97–2.04)	0.076
50+	0.33 (0.17–0.60)	<0.001	0.43 (0.31–0.60)	<0.001	0.69 (0.52–0.91)	0.010	2.58 (1.52–4.32)	<0.001
Sex								
Female	—		—		—		—	
Male	1.65 (1.16–2.38)	0.007	1.13 (0.94–1.35)	0.2	0.45 (0.38–0.52)	<0.001	3.28 (2.18–5.12)	<0.001
Testing type								
Targeted testing	—		—		—		—	
Population-based screening	1.32 (0.89–1.96)	0.2	0.40 (0.32–0.50)	<0.001	1.08 (0.89–1.31)	0.4	0.35 (0.21–0.54)	<0.001
Region								
West Africa	—		—		—		—	
East Africa	0.86 (0.54–1.36)	0.5	1.03 (0.83–1.27)	0.8	0.89 (0.73–1.09)	0.3	0.46 (0.30–0.71)	<0.001
Southern Africa	2.81 (1.91–4.12)	<0.001	1.98 (1.57–2.49)	<0.001	0.82 (0.66–1.01)	0.062	1.11 (0.72–1.68)	0.6

Abbreviations: HBeAg, hepatitis B e-antigen; HBV, hepatitis B virus; OR, odds ratio; TE, transient elastography.

a HBV DNA dichotomised at <2,000 vs. ≥2,000 IU/ml.

b ALT dichotomised at <30 vs. ≥30 IU/L (men) and <19 vs. ≥19 IU/L (women).

c Cirrhosis was dichotomised based on TE <9.5 (no) vs. ≥9.5 kPa (yes).

## Discussion

HEPSANET is a unique research collaboration that was established to close policy-relevant data gaps, catalyse progress towards HBV elimination, and strengthen research capacity in Africa. We here describe the genesis of the collaboration, its current structure, scientific priorities, and the baseline characteristics of included participants living with HBV across all HEPSANET sites.

HEPSANET includes a diverse sample from various parts of Africa. Most contributing cohorts are managed at hospitals staffed by local experts; however, the majority of participants learned their HBV status from routine screening approaches and entered HEPSANET before treatment. Data from such individuals (i.e. diagnosed before the onset of advanced liver disease or HBV complications) may be more representative of the general population with HBV in this region than people diagnosed when they present with signs or symptoms. Also, a major strategy for HBV elimination is identifying people early and linking them to care. Given that most entered HEPSANET asymptomatically and untreated, this will allow HEPSANET to evaluate existing indications to start antiviral therapy and potentially develop new treatment criteria based on local data. Similar to other health conditions, men were slightly more likely than women to learn their HBV status once they developed signs or symptoms.

The collaboration is also well positioned to analyse HBV natural history in Africa because a range of HBV phenotypes have been described. Among adults in HEPSANET, HBeAg-positivity was relatively rare; hence, few were classified as having HBeAg-positive chronic HBV infection. The rarity of HBeAg-positive chronic HBV infection supports the notion that HBeAg seroconversion happens in adolescence and early adulthood in Africa. Other HBV phenotypes were well represented, including with HBeAg-negative chronic hepatitis B, a predominant phenotype in Africa that is associated with liver disease progression, possibly more than HBeAg-positive hepatitis [27]. Building on prior data [28], we also described that 42.1% could not be categorised, mostly due to elevated ALT in the setting of HBV DNA <2,000. A better understanding of the causes of ALT elevation in these individuals, such as unhealthy alcohol use [29] or metabolic dysfunction-associated fatty liver disease, is needed. Many real-world clinics in Africa lack access to HBV DNA testing and have to rely on ALT to make treatment decisions [20].

In this analysis, we also detected some possible regional differences in HBeAg and HBV DNA, which is an important finding that could be explained by Africa’s diversity in terms of environmental co-factors, host genetics, and circulating HBV genotypes. Few studies have compared the clinical significance of circulating genotypes in Africa, the way it has been done in Asia for genotypes B and C. In the Gambia, among 271 untreated participants with chronic HBV infection and known genotype, a similar proportion with genotypes E and A were treatment-eligible [30]. HEPSANET will be poised to better understand these issues through longitudinal analysis. Although we excluded participants who were symptomatic when diagnosed and adjusted for age and sex, there may be unaddressed factors confounding in the relationship between region and markers of HBV replication and liver disease.

The major strength and unique feature of HEPSANET is the ability to harmonise good quality data in a large sample of people living with hepatitis B in Africa. Observed differences in HBV and liver markers by region strongly support the need for a multi-regional approach. HEPSANET also has a good representation of sex and adult age groups, which are two of the strongest factors driving HBV natural history, and, while most were HBeAg-negative, pooled data will permit well-powered analyses of participants with HBeAg-positive phenotypes.

Another strength of HEPSANET is that harmonised data will generate statistical power to analyse rare outcomes (like cirrhosis progression, HCC incidence, and HBsAg seroclearance) and the ability to analyse HBV patient groups that have been underrepresented in past studies, such as individuals aged over 50 years and those with chronic alcohol use and chronic metabolic disorders. A final and important strength is our access to untreated participants, which will allow HEPSANET to evaluate treatment eligibility and the clinical outcomes of treated and untreated subjects and potentially become a platform for clinical trials in the future. At HBV centres of excellence in upper-income settings, most patients with chronic HBV infection are either inactive or on treatment.

There are also potential weaknesses to HEPSANET. First, we face heterogeneity across sites in terms of recruitment procedures. Future research will improve the standardisation and classification of these procedures, for instance, by distinguishing between participants recruited from antenatal clinics and those referred following routine screening in other settings. Treatment initiation is based on local guidelines, and in countries with high HIV burden, HBV mono-infection is occasionally treated with two anti-HBV active drugs instead of the recommended single drug use. Such heterogeneity will be acknowledged and addressed when possible using advanced approaches to observational cohort analysis. Second, laboratory assays are also different across sites, which could introduce some bias. As HEPSANET matures, it is intended that data collection procedures will be harmonised wherever possible. Finally, unlike cohorts of people with HBV in upper-income countries where out-of-pocket payment by patients for long-term follow-up is not very important, national hepatitis programmes in Africa have limited funding to subsidise HBV testing and treatment to make them accessible to wider population. Consequently, the majority of people living with chronic HBV infection, except for a few covered by health insurance or enrolled in a research project, must pay important user fees to get access to laboratory tests required for the initial assessment and follow-up visits. This indicates a difficulty in obtaining a true picture representing the general population in this region.

### Scientific agenda and future directions

HEPSANET represents the largest group of hepatitis B researchers in Africa and has, through consensus, established research priorities for hepatitis B in Africa. The collaboration’s research strategies going forward will be based on these priorities. For example, improving the understanding of the natural history of chronic HBV infection was the highest-ranked priority. HEPSANET’s primary focus is currently to support longitudinal follow-up of well-defined cohorts. This includes the analysis of both treated and untreated infections and will include data from additional hepatitis B cohorts that have been invited to participate but have not yet entered their baseline data. The majority of resources required to establish longitudinal follow-up will be dedicated to strengthening data collection and management. This also includes providing resources for cohort staff on the ground with a goal to improve the quality of data (e.g. less missing data) and duration of follow-up of patients (e.g. improve retention). It is expected that HEPSANET will continue long term as long as the scientific and public health need endures. While future grant funding will be required to scale up the size of HEPSANET and strengthen administration functions, core procedures are mostly funded externally and can continue independently of additional resources.

HEPSANET’s second priority, to develop simplified HBV treatment eligibility criteria for Africa, is being addressed through several current analyses. In one recent publication, a new threshold for the AST-to-platelet ratio index was proposed based on HEPSANET data [25]. Defining best practices for HBV testing and linkage to care is represented in several top-10 priorities. Going forward, HEPSANET will expand data collection around HBV testing and downstream care in order to identify best practices and gaps in the HBV care continuum. Finally, there is also interest in creating sentinel sub-cohorts within HEPSANET to facilitate more in-depth analysis of certain issues. For example, a future sub-cohort would facilitate HBV translational science through structured sample collection and storage with virology, immunology, and biomarker tests done at a central laboratory. Another sub-cohort may focus on the social and behavioural elements of HBV in Africa including stigma and discrimination and behavioural economics [31, 32].

## Conclusion

HEPSANET is a unique transnational continental research cohort collaboration that was created to close data gaps in the area of HBV in Africa. To date, eight countries and more than 4,000 people with chronic HBV infection have been included and analysed. The long-term vision is to use team science and synergy across cohorts and between diverse investigators to address the most pressing questions in the field. This will ensure that the people of Africa, and crucially those living with HBV, are represented in global elimination strategies, and facilitate the elimination of HBV as a public health threat in Africa.

## Data Availability

The data that support the findings of this study are available from the corresponding author upon reasonable request.

## Supplementary material

For supplementary material accompanying this paper visit http://doi.org/10.1017/S095026882300050X. click here to view supplementary material
