# Supplementary material for: Hepatitis B in Africa Collaborative Network: cohort profile and analysis of baseline data
Source: Epidemiol Infect. 2023 Apr 3;151:e65. doi: 10.1017/S095026882300050X (PMC10204139; doi:10.1017/S095026882300050X)
Supplement: Supplementary file 1 [file S095026882300050Xsup001.docx]

| **Supplementary table 1: Core and enhanced variables being collected in HEPSANET dataset** | |
| --- | --- |
| **DATA TYPE** | **VARIABLES** |
| **Core administrative and demographic data** | Unique client ID, date of enrollment, age, sex, pregnancy status |
| **Core treatment status** | Current HBV treatment status, treatment start date, treatment stop date (if applicable), medication names |
| **Core clinical and laboratory data** | *Core diagnostic data*: Initial HBsAg test date, HIV test date and results,  *Core lab data*: ALT date and level, AST date and level, platelet count date and level  *Core physical exam data*: Physical exam date, physical exam evidence of cirrhosis / abdominal mass, past or current evidence of decompensated liver disease, past or current evidence of HCC  *History of HCC:* HCC date of diagnosis, HCC modality of diagnosis  *Pregnancy:* Last menstrual period, pregnancy outcome, type of delivery, infant birth weight, infant HBV birth dose, infant HBIG, infant additional HBV vaccines, infant HBsAg test date and results |
| **Core cohort outcome data** | Final cohort outcome, date the clinic/program last communicated with patient, date of withdrawal (if applicable), date of transfer out (if applicable), date of death (if applicable) |
| **Enhanced clinical and laboratory data** | *Diagnosis data:* Reason for HBV testing, HBV diagnosis data  *Physical measurements*: Height, weight  *Liver cofactors:* Diabetes, steatosis, schistosomiasis, alcohol use status, use of herbal medicine, use of Khat, use of other substances,  *Expanded laboratory data:* HBV DNA, HBeAg, GGT, total bilirubin, PT/INR, alpha fetoprotein, hepatitis delta antibody, hepatitis C antibody, liver elastography, abdominal ultrasound, |
| **Expanded cohort outcome data** | Cause of death, type of liver related death, source of information on death |
